# Incidence of Upper Extremity Neuropathies at a Single Tertiary Care Institution in the United States: Trends in Recent Years, Including the COVID-19 Pandemic

**DOI:** 10.7759/cureus.61458

**Published:** 2024-05-31

**Authors:** Taylor F Faust, Megan R Donnelly, Akram Habibi, Pablo Castaneda

**Affiliations:** 1 Department of Research, Alabama College of Osteopathic Medicine, Dothan, USA; 2 Department of Orthopedic Surgery, NYU Langone Health, New York, USA; 3 Department of Pediatric Orthopedic Surgery, Baylor Scott & White George Truett James Orthopaedic Institute, Houston, USA

**Keywords:** northeast united states, orthopedics, ulnar nerve neuropathy, carpal tunnel syndome, upper extremity neuropathy

## Abstract

Objective: This study examined trends in upper extremity (UE) neuropathies at a large urban tertiary care center in the Northeastern United States over the past five years, including the period of the COVID-19 pandemic.

Method: A retrospective medical record review was conducted from 2018 to 2022. We collected data from unique patient records identified using International Classification of Diseases, Tenth Revision (ICD-10) codes for UE neuropathies. We characterized subjects by age, demographics, and duration of symptoms.

Results: This study included 288 pediatric patients and 51,997 adult patients newly diagnosed with UE neuropathy. Most patients were aged 55+; 0.4% of all patients diagnosed with UE neuropathy were children. Across all ages, there was an overall increase in UE neuropathy diagnoses in the past five years, with the most noticeable increases from 2018 to 2019 (+5,761 diagnosed individuals, or +122%) and from 2020 to 2021 (+2,769 diagnosed individuals, or +28.8%).

Conclusion: Our institution's UE neuropathy diagnoses have increased in the past five years. Of note, there was a significantly increased rate of UE neuropathy diagnoses from 2020 to 2021. This increase coincides with the COVID-19 pandemic, which is leading to a changing environment for many Americans. These societal changes will likely become indelible after the pandemic; safety practices should be enacted to avoid these debilitating neuropathies.

## Introduction

Upper extremity (UE) neuropathies affect millions of individuals worldwide every year [1]. In fact, one of the most common orthopedic conditions is carpal tunnel syndrome (CTS), which has an estimated 2.8 million new cases diagnosed annually [2]. Given the significant burden, these UE neuropathies place on the patient and the healthcare system, the etiology and epidemiology of these conditions have been thoroughly investigated. 

Orthopedic literature has reported many risk factors for different UE neuropathies. For example, an often-hypothesized, although highly controversial, risk factor for CTS involves repetitive microtrauma at the level of the wrist, which may stem from spending excessive time each day at the computer, typing on a keyboard, and using a mouse with certain unnatural hand positions [3,4]. Additionally, uncontrolled or poorly controlled diabetes can lead to the sequelae of diabetic neuropathy. In these patients, microangiopathic changes at the level of the nerve can lead to decreased myelination and nerve fiber loss, often causing irreversible changes [5-7]. Finally, other systemic diseases, trauma, tumors, and metabolic diseases can all contribute to developing UE neuropathy [8].

Notably, it is known that a subset of these risk factors became more prevalent in society in the wake of the novel COVID-19 pandemic. For instance, one of the significant changes brought about by the pandemic was the implementation of stay-at-home orders. Requiring people to work from home likely encouraged individuals to lead a more sedentary lifestyle, spending more time at the computer with poor posture and upper extremity positioning and dedicating less time to being active. As such, it can be inferred that at least two risk factors for UE neuropathies increased: the spike in microtrauma from increased computer time and an increased prevalence of diabetes or metabolic disease attributed to overall inactivity [9]. Furthermore, during the COVID-19 pandemic, there was an increase in critical care patients. A known complication of critical care is polyneuropathy [10], due to direct compression or traction of the plexus, peripheral nerves, or arterial structures [11]. Thus, the COVID-19 pandemic likely influenced the diagnosis rate in UE neuropathy patients, as it created the perfect storm of factors associated with the etiology of this complex disease.

The trends in UE neuropathies in the United States during the pandemic need to be better characterized, as this period represented a truly unprecedented time for the healthcare system. This study aimed to evaluate the trends in UE neuropathy incidence over the past five years, including during the COVID-19 pandemic, at a single large, urban tertiary care facility in the United States. In the context of the increase in working from home and the diagnosis of metabolic diseases such as diabetes, we hypothesized that there would be an increasing rate of UE neuropathy diagnoses as the COVID-19 pandemic reshaped society and forever altered public health in America.

## Materials and methods

After institutional review board approval was waived, we retrospectively reviewed the electronic medical record system at NYU Langone Health in New York City, a single, large, tertiary care medical center, from 2018 to 2022. We identified all patients at our institution with a new diagnosis of UE neuropathy, such as CTS, cubital tunnel syndrome, thoracic outlet syndrome (TOS), and Guyon canal syndrome, using the International Classification of Diseases, Tenth Revision (ICD-10 codes) (Appendix A). No funding was provided for this study.

Subsequently, UE neuropathy patients were divided by year of diagnosis and by age group. The age groups included were under 18, 18-24, 25-34, 35-44, and 55+ years or older. Using Microsoft Excel (Microsoft Corp., Redmond, WA), we created scatterplots to display the yearly trends in UE neuropathy diagnosis. This data determined the aggregate numbers of patients seen at our institution by year and age group. Finally, to calculate the rate of UE neuropathy diagnoses annually at our institution, the total number of UE neuropathy diagnoses was weighted by the total number of patients seen at our institution per year per 100,000 individuals.

## Results

We identified 228 newly diagnosed pediatric patients and 51,997 newly diagnosed adult patients with UE neuropathy at our institution from 2018 to 2022. The majority of patients diagnosed with neuropathy were aged 55+ years (56.7%), followed by those in the age group of 45-54 years (19.2%) and those in the age range of 35-44 (12.9%). Only around 0.4% of all patients diagnosed with UE neuropathy in the past five years were children.

There was an overall increase in new patients diagnosed with UE neuropathy across all age groups during this time, with the most noticeable increases from 2018 to 2019 (+5,761 diagnosed individuals, or +122%) and from 2020 to 2021 (+2,769 diagnosed individuals, or +28.8%). Although there were few pediatric patients, this trend was similar to that of adults, with an increase of +59.1% in UE neuropathy diagnoses from 2018 to 2019 and +42.2% in diagnoses from 2020 to 2021 (Table 1).

**Table 1:** Number of new diagnoses of upper extremity (UE) neuropathies in the past five years * Data included from 1/1/2022 to 12/21/2022

	Year					
Age (in years)	2018	2019	2020	2021	2022*	Total
Under 18	22	35	45	64	62	228
18-24	96	263	200	261	249	1069
25-34	442	934	842	1107	1192	4517
35-44	640	1349	1278	1606	1890	6763
45-54	971	2017	1891	2414	2743	10036
55+	2553	5887	5348	6921	8903	29612
Total	4724	10485	9604	12373	15039	52225

Regarding the total number of patients seen at our facility during the past five years, there were 2,576,426 pediatric patients and 24,477,201 adult patients. In sum, the medical center documented 27,053,627 patients. Throughout the study period, the total number of patients increased from 2018 to 2022 (+1,295,075 patients, or +176.7%) (Table 2).

**Table 2:** Total number of patients seen at our facility over the past five years * Data included from 1/1/2022 to 12/21/2022

	Year					
Age (in years)	2018	2019	2020	2021	2022*	Total
Under 18	305,055	677,505	568,143	656,783	368,940	2,576,426
18-24	111,053	253,408	264,985	331,799	171,652	1,132,897
25-34	270,548	627,712	685,739	875,662	435,997	2,895,658
35-44	297,600	694,182	720,489	922,913	473,662	3,108,846
45-54	374,883	846,066	830,728	1,030,238	530,517	3,612,372
55+	1,414,986	3,250,997	3,114,031	3,858,982	2,088,432	13,727,428
Totals	2,774,125	6,349,810	6,184,113	7,676,377	4,069,200	27,053,627

**Figure 1:**
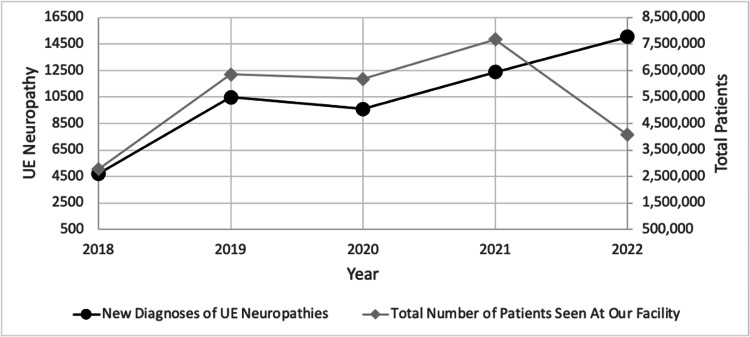
Trends in new diagnoses of upper extremity (UE) neuropathies and total patients seen at our facility in the past five years

The scatterplot demonstrates the steady increases in newly diagnosed UE neuropathy patients and total patients seen at our institution annually (Figure 1).

When we weighted the annual number of newly diagnosed UE neuropathies by the total number of patients seen at our institution per 100,000 individuals, the overall rates of UE neuropathies were also demonstrated to increase, with the most dramatic increase in 2022 compared to 2021 (+129.3% increase). The increased rate was seen consistently across all adult age groups (Table 3).

**Table 3:** Rate of new diagnoses of upper extremity (UE) neuropathy weighted by the total number of patients seen at our facility per 100,000 individuals * Data included from 1/1/2022 to 12/21/2022

	Year					
Age (in years)	2018	2019	2020	2021	2022*	Total
Under 18	7.21	5.17	7.92	9.74	16.80	8.85
18-24	86.45	103.79	75.48	78.66	145.06	94.36
25-34	163.37	148.79	122.79	126.42	273.40	155.99
35-44	215.05	194.33	177.38	174.01	399.02	217.54
45-54	259.01	238.40	227.63	234.31	517.04	277.82
55+	180.43	181.08	171.74	179.35	426.30	215.71
Totals	170.29	165.12	155.30	161.18	369.58	193.04

**Figure 2:**
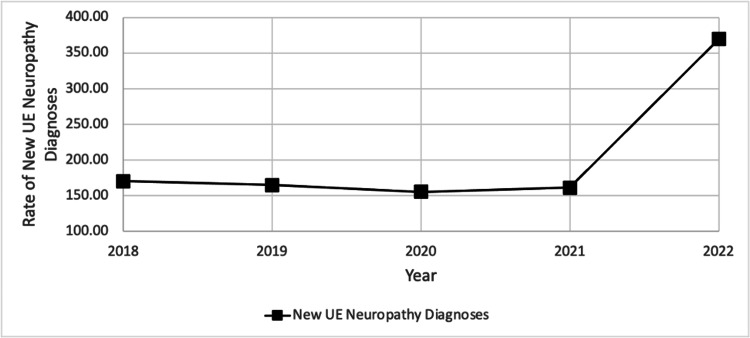
Rate of new diagnoses of upper extremity (UE) neuropathy weighted by the total number of patients seen at our facility per 100,000 individuals

The increased rate of newly diagnosed UE neuropathies, particularly in 2021 and 2022, is displayed clearly in the scatterplot (Figure 2).

## Discussion

The increasing prevalence of UE neuropathies in recent years has brought attention to the potential impact of societal changes on the healthcare system. Millions of individuals worldwide are diagnosed with these conditions annually, and the pandemic's unique circumstances may have contributed to this rise. We will explore the key findings of our study, highlighting the potential factors that could be associated with the increased diagnosis rates. While our study cannot establish causation, these correlations are worth investigating further to develop effective prevention strategies.

The COVID-19 pandemic reshaped the socioeconomic landscape, with more people working from home and experiencing a sedentary lifestyle, potentially leading to metabolic diseases such as diabetes and other risk factors for UE neuropathies. Our study at a single large urban tertiary care center in the US showed a nearly 30% increase in diagnosis rates from 2020 to 2021 across all patients. Pediatric patients saw an even steeper 42% increase. While we cannot conclusively attribute these changes to the pandemic, the temporal coincidence suggests a potential connection.

This study investigated some of the most common UE neuropathies affecting the median, ulnar, radial, axillary, and musculocutaneous nerves of the arm. These neuropathies were categorized based on the location of injuries, either the upper arm or the forearm, wrist, or hand. We then identified specific lesions affecting the nerves themselves, utilizing the ICD codes that describe and classify them. Notable conditions include CTS, cubital tunnel syndrome, TOS, and Guyon's canal syndrome.

Carpal tunnel syndrome is a condition that involves the compression of the median nerve at the distal forearm, where it meets the wrist within the carpal tunnel. The symptoms include numbness, tingling, weakness, and pain in the hand, thumb, index finger, middle finger, and half of the ring finger [12]. Cubital tunnel syndrome is a neuropathy due to the compression of the ulnar nerve at the elbow at the medial epicondyle. The associated symptoms include numbness, tingling, weakness, and pain in the hand, particularly affecting the hypothenar side, the fourth finger, and the fifth finger [12]. Thoracic outlet syndrome is a more nonspecific diagnosis that encompasses a collection of conditions involving compression of neurovascular structures at the thoracic outlet, which consists of the first rib, the scalene muscles, and the clavicles. Symptoms may include pain, tightness, weakness, and numbness extending from the hand to the shoulder and can even include the neck [12]. Guyon’s canal syndrome is an ulnar nerve neuropathy at the distal wrist, where the ulnar nerve is compressed before it enters the hand. The associated symptoms include weakness, paralysis, numbness, and tingling in the hypothenar side of the hand, specifically affecting the ring and pinky fingers [13]. While these examples represent only a portion of the UE neuropathies discussed, other neuropathies can also affect the same or different nerves of the upper extremity, leading to various types of impairment.

Several factors might contribute to the surge in UE neuropathies. Our study focused on working-aged adults [14], and we hypothesized that the more sedentary lifestyle associated with remote work might be linked to poor posture and compression of UE nerves. Studies have shown associations between computer use and peripheral nerve impairments, supporting this idea [15]. While these findings merit the hypothesis, further research is necessary to fully understand the underlying mechanisms.

Another consideration is the potential role of the coronavirus infection itself. The novel coronavirus has been known to trigger an inflammatory cascade in the body, historically associated with neuropathies [16]. Critical illness and prolonged periods of immobilization, as seen in some COVID-19 patients, could increase the risk for neuropathies. While our study cannot directly link COVID-19 infection to UE neuropathy diagnoses, it warrants further investigation.

Surprisingly, pediatric patients also showed increased UE neuropathy diagnoses during the pandemic [17]. Extended hours spent at home studying in a virtual setting without regular breaks may have led to poor posture and compression of nerves [18]. The rising rates of obesity and diabetes in children during the pandemic may have contributed to this increase. Jenssen et al. found that the overall obesity rate of children between the ages of two and 17 had increased from 13.7% to 15.4% from December 2019 to December 2020 [19]. Interventions tailored to this population are crucial to addressing these potential risk factors. An already established association between CTS and obesity highlights how an increase in body mass index (BMI) can lead to the slowing of nerve conduction, resulting in a higher incidence of this neuropathy [20]. Seror et al., who looked at bilateral median nerve lesions at the wrist and their connections to obesity and diabetes, found that their prevalence was higher in obese patients with diabetes than in the general population [21]. This link between obesity and median nerve deficiencies in individuals with diabetes highlights the need for further research into the impact of high BMI and the biochemical changes in glucose metabolism on other UE neuropathies.

The significance of this study lies in its insights into the prevalence of UE neuropathies, the possible consequences of the COVID-19 pandemic, and how societal shifts may affect our healthcare system. With millions diagnosed with UE neuropathies each year, understanding these trends is vital for healthcare providers to implement prevention measures effectively and adapt their treatment strategies. By emphasizing early intervention, education, and tailoring safety practices, providers can help alleviate the impact of these debilitating conditions. 

We acknowledge the limitations of our study, such as its retrospective nature and the possibility of misdiagnosis and missing information in medical records. Furthermore, our study focused on a specific urban population, limiting the generalizability of the results to the entire country. Future research should consider longer study periods and include more diverse populations to better understand the epidemiological risk factors for UE neuropathy.

## Conclusions

Upper extremity neuropathies represent a significant and increasing healthcare burden, possibly exacerbated by societal changes brought on by the COVID-19 pandemic. While we cannot definitively establish causation, our study's findings warrant further investigation into potential risk factors and preventive measures. Early intervention, education, and safety practices, especially for those working from home, may help mitigate the impact of these debilitating conditions. Addressing these issues will require interdisciplinary collaboration and tailored interventions, particularly for pediatric patients, to mitigate the long-term effects of the pandemic on UE neuropathy rates.

## Appendices

Appendix A 

**Table 4:** New diagnoses of upper extremity (UE) neuropathy, such as carpal tunnel syndrome, cubital tunnel syndrome, thoracic outlet syndrome, and Guyon canal syndrome, using the International Classification of Diseases, tenth revision (ICD-10 codes)

ICD Codes	Descriptor (includes unspecified, unilateral left, unilateral right, bilateral variations)
G56.0	Carpal tunnel syndrome
G56.1	Other lesions of the median nerve
G56.2	Lesions of the ulnar nerve
G56.3	Lesions of the radial nerve
G56.4	Causalgia of the upper limb
G56.8	Other specified mononeuropathies of the upper limb
G569	Unspecified mononeuropathies of the upper limb
S44.0	Injury of the ulnar nerve at the upper arm level
S44.1	Injury of the median nerve at the upper arm level
S44.2	Injury of the radial nerve at the upper arm level
S44.3	Injury of the axillary nerve
S44.4	Injury of the musculocutaneous nerve
S44.5	Injury of the cutaneous sensory nerve at the shoulder and upper arm level
S44.8	Injury of other nerves at the shoulder and upper arm level
S44.9	Injury of unspecified nerve at shoulder and upper arm level
S54.0	Injury of the ulnar nerve at the forearm level
S54.1	Injury of the median nerve at the forearm level
S54.2	Injury of the radial nerve at the forearm level
S54.3	Injury of the axillary nerve at the forearm level
S54.8	Injury of other nerves at the forearm level
S54.9	Injury of an unspecified nerve at the forearm level

## References

[1] Wahab KW, Sanya EO, Adebayo PB, Babalola MO, Ibraheem HG (2017). Carpal tunnel syndrome and other entrapment neuropathies. Oman Med J.

[2] Einhorn N, Leddy JP (1996). Pitfalls of endoscopic carpal tunnel release. Orthop Clin North Am.

[3] Gupta R, Rummler L, Steward O (2005). Understanding the biology of compressive neuropathies. Clin Orthop Relat Res.

[4] Doughty CT, Bowley MP (2019). Entrapment neuropathies of the upper extremity. Med Clin North Am.

[5] Tracy JA, Dyck PJ (2008). The spectrum of diabetic neuropathies. Phys Med Rehabil Clin N Am.

[6] Malik RA, Tesfaye S, Newrick PG (2005). Sural nerve pathology in diabetic patients with minimal but progressive neuropathy. Diabetologia.

[7] Yagihashi S (1995). Pathology and pathogenetic mechanisms of diabetic neuropathy. Diabetes Metab Rev.

[8] Kabakçi ADA (2021). Upper extremity entrapment neuropathy. Demyelination Disorders.

[9] Rydberg M, Zimmerman M, Gottsäter A, Nilsson PM, Melander O, Dahlin LB (2020). Diabetes mellitus as a risk factor for compression neuropathy: a longitudinal cohort study from southern Sweden. BMJ Open Diabetes Res Care.

[10] Lacomis D (2011). Neuromuscular disorders in critically ill patients: review and update. J Clin Neuromuscul Dis.

[11] Miller C, O'Sullivan J, Jeffrey J, Power D (2021). Brachial plexus neuropathies during the COVID-19 pandemic: a retrospective case series of 15 patients in critical care. Phys Ther.

[12] Thatte MR, Mansukhani KA (2011). Compressive neuropathy in the upper limb. Indian J Plast Surg.

[13] Kaplan J, Kanwal A (2024). Thoracic Outlet Syndrome. https://www.ncbi.nlm.nih.gov/books/NBK557450/.

[14] (2023). Employment status of the civilian noninstitutional population by age, sex, and race. https://www.bls.gov/cps/cpsaat03.htm.

[15] Schmidt SC, Anedda B, Burchartz A (2020). Physical activity and screen time of children and adolescents before and during the COVID-19 lockdown in Germany: a natural experiment. Sci Rep.

[16] Oaklander AL, Mills AJ, Kelley M, Toran LS, Smith B, Dalakas MC, Nath A (2022). Peripheral neuropathy evaluations of patients with prolonged long COVID. Neurol Neuroimmunol Neuroinflamm.

[17] Giorda CB, Gnavi R, Tartaglino B (2023). Increased incidence of type 1 diabetes in 2 years of COVID-19 pandemic. Acta Diabetol.

[18] Jepsen JR (2004). Upper limb neuropathy in computer operators? A clinical case study of 21 patients. BMC Musculoskelet Disord.

[19] Jenssen BP, Kelly MK, Powell M, Bouchelle Z, Mayne SL, Fiks AG (2021). COVID-19 and changes in child obesity. Pediatrics.

[20] Nathan PA, Keniston RC, Myers LD, Meadows KD (1992). Obesity as a risk factor for slowing of sensory conduction of the median nerve in industry. A cross-sectional and longitudinal study involving 429 workers. J Occup Med.

[21] Seror P, Seror R (2013). Prevalence of obesity and obesity as a risk factor in patients with severe median nerve lesion at the wrist. Joint Bone Spine.

